# Around the Hospitals

**Published:** 1896-10-10

**Authors:** 


### AROUND THE HOSPITALS.

[Notice to the Managers of Institutions.—Special space is now reserved for the insertion of notices of hospital meetings and festivals. The Editor requests that all notices may reach The Hospital Office, 28 & 29, Southampton Street, Strand, London, W.C., at least one week before the date of each meeting.]

**St. Thomas's Hospital.**—The second annual report of the treasurer, recently issued, opens by insisting on the disparity of the hospital accommodation in relation to population which exists on the south side of the Thames compared with that found on the north side. Any increase in the number of hospitals is, however, deprecated on the ground that Guy's and St. Thomas's have still between them room for nearly 200 more patients, which could be utilised did funds permit. As our readers know, two of the long-closed wards at St. Thomas's were opened in February, 1896, and in natural sequence this has rendered an increase in the nursing staff a necessity. To accommodate these nurses and also those who had formerly occupied several of the rooms attached to the re-opened wards, the old apothecary's house has been fitted up as a nurses' home, providing rooms for four sisters and twelve nurses. The opportunity for making changes in connection with the sanitary and domestic conveniences of the wards afforded by their preparation for the reception of patients was taken advantage of, and the lighting arrangements were carefully considered. Each bed has now been given an independent electric light, while wall sockets for hand electric lamps have rendered the use of candles or paraffin lamps in these wards unnecessary. A change in the working of part of the hospital was made on the retirement of Mr. Walker, the late steward, the steward's duties being rearranged and a lady housekeeper appointed. The latter, under the superintendence of the matron, now has charge of all female labour, is responsible for the cooking of all food, and generally supervises the cleaning of all portions of the hospital other than the wards. The statistics given in the report show that during the last four years the in-patients admitted have steadily increased, the admissions being 5,409 in 1892 and 5,780 in 1895, while the average number of days each patient has resided in the hospital has as steadily decreased. Indeed this latter item has decreased during the last ten years, there being a decrease year by year, with one exception (27·5 in 1887) from 27·17 days in 1886 to 21·72 days in 1895. The out-patients (including 92,675 casualties) amounted during 1895 to 114,627. As showing the vast amount of work done at St. Thomas's Hospital we may mention in conclusion that exclusive of the 2,571 maternity cases, who were visited at their own homes, but who are included among the number just given, 153,724 attendances were registered by the out-patients, or an average of 491 persons seen in the out-patient department per day, and adding to this the average daily number of in-patients treated in the wards (366) we find that the large number of nearly 860 patients are treated at St. Thomas's Hospital every day.

